# Pregnancy outcome in patients with neuromyelitis optica spectrum disorder treated with rituximab: A case-series study

**DOI:** 10.22088/cjim.12.0.491

**Published:** 2021

**Authors:** Maral Seyed Ahadi, Mohammad Ali Sahraian, Vahid Shaygannejad, Nassim Anjidani, Seyed Ehsan Mohammadiani Nejad, Nahid Beladi Moghadam, Hormoz Ayromlou, Gholam Ali Yousefi Pour, Sepideh Yazdanbakhsh, Mehrdad Jafari, Abdorreza Naser Moghadasi

**Affiliations:** 1 Multiple Sclerosis Research Center, Neuroscience Institute, Tehran University of Medical Sciences, Tehran, Iran; 2 Isfahan Neurosciences Research Center, Isfahan University of Medical Sciences, Isfahan, Iran; 3 Medical Department, Orchid Pharmed Company, Tehran, Iran; 4 Department of Neurology, Ahvaz Jundishapur University of Medical Sciences, Ahvaz, Iran; 5 Department of Neurology, Shahid Beheshti University of Medical Sciences, Tehran, Iran; 6 Department of Neurology, Faculty of Medicine, Tabriz University of Medical Sciences, Iran; 7 Department of Neurology, Shiraz University of Medical Sciences, Shiraz, Iran; 8 Imam Khomeini Hospital complex, Tehran University of Medical Sciences, Tehran, Iran

**Keywords:** Neuromyelitis optica spectrum disorder, Rituximab, Pregnancy

## Abstract

**Background:**

Neuromyelitis optica spectrum disorder (NMOSD) is a neuroinflammatory disorder with a tendency to affect the spinal cord and optic nerves. As NMOSDs have a predilection for women of reproductive age and adopt an aggressive course during pregnancy, appropriate treatment strategies before conception and during pregnancy should be well-considered.

**Case Presentation:**

In this report, the pregnancy outcome of eight pregnancies following rituximab treatment was assessed, which led to 50% live births with mean birth weight of 2777.50 (SD: 545.92) grams. Two patients had abortions due to doctor’s recommendation. One pregnancy led to intrauterine fetal death (IUFD) due to nuchal cord. No spontaneous abortions were encountered. Two patients received rituximab during pregnancy. No major malformations or serious neonatal infections were encountered.

**Conclusion:**

Rituximab should be administered by caution in NMOSD patients who want to be pregnant and the probable adverse effects of the drug should be discussed by patients.

Neuromyelitis optica spectrum disorder (NMOSD) is a neuroinflammatory disorder with a propensity to affect the spinal cord and optic nerves. As NMOSDs have a predilection for women of reproductive age and adopt an aggressive course during pregnancy (1), appropriate treatment strategies before conception and during pregnancy should be well-considered. Novel therapeutics targeting CD20+ B cells are increasingly incorporated in NMOSD due to considerable annualized relapse rate reduction and disability improvement (2, 3). However, their administration in pregnancy is limited due to safety concerns. As this drug has a long half-life (approximately 3 weeks), this study can be helpful (4). This is a report of eight NMOSD patients followed from Nov 2017 to May 2020, who have conceived during rituximab treatment. Data were collected prospectively from the NMOSD registry of Sina Hospital, a tertiary neuroinflammatory center in Tehran. Pregnant patients were enrolled, and a standardized questionnaire enquiring about their clinical and gynecological characteristics was completed. Thereafter, patients were visited once in each trimester during pregnancy and a month after childbirth for pregnancy outcome, disease activity, and lactation status. Zytux^TM ^(rituximab, AryoGen Pharmed), a biosimilar form of rituximab proven to be equally effective as MabThera® (5, 6), was administered 1g twice, two weeks apart initially in NMOSD patients and 1g every six months thereafter. This study was approved by the Ethics Committee of Tehran University of Medical Sciences (approval number IR.TUMS.VCR.REC.1397.322). 

## Case presentation

Eight patients with a mean age of 33 (SD: 4.21) were enrolled in the study. Table 1 depicts the patients’ clinical and demographic characteristics.

**Table 1:** Clinical and Demographic Characteristics of Patients

*Patient*	Age	Disease Duration (y)	Pregnancy After Rituximab Initiation (m)	The numbers of Rituximab injections	Interval between last Rituximab injection and pregnancy (m)	Gestation	Abortion History	Pregnancy Outcome	Gestational Week	Birth Weight (g)	Rituximab Administration During Pregnancy	Rituximab Administration After Delivery (d)
*1*	33	9	8.87	2	2.33	1	No	Live Birth	41w+6d	3110		10
*2*	31	3	6.70	1	0.50	2	Yes	Live Birth	38w	2800		10
*3*	29	4	15.41	3		1	No	Abortion	5			
*4*	28	4	6.83	1	0.33	1	No	Live Birth	40w+ 4d	2000		
*5*	38	14	15.31	3	2.70	2	No	Still Birth	36		30^th^ week of pregnancy	
*6*	39	12	15.97	3	2.50	3	No	Live Birth	43w +2d	3200	17^th^ week of pregnancy	33
*7*	30	5	16.99	3	4.00	4	Yes	Abortion	8			
*8*	36	3	4.90	1	4.90	1	No	Ongoing				

Patients conceived 11.37 (SD: 5.00) months after the start of rituximab and were followed for a mean of 25.45 (SD: 15.07) months. Patients were under treatment with rituximab based on the national guidelines (7). Two patients had abortions, according to the doctor’s recommendation. Six patients continued pregnancy to the full term of whom one pregnancy led to intrauterine fetal death (IUFD) due to nuchal cord at the 37th of gestation. Four term live births were reported, and one pregnancy was ongoing at the time of writing this report. The delivery route choice was determined based on gynecological indications. The offspring consisted of two females and two males with a mean birth weight of 2777.50 (SD: 545.92) grams. No major malformations or serious neonatal infections were reported during the follow-up period. Routine national vaccination program was performed in all the babies. 

Two patients received the maintenance dose of rituximab during pregnancy (500 and 100 mg) based on treating the physicians’ recommendations, and no maternal complications were encountered. However, the pregnancy treated with 500 mg rituximab was terminated due to the nuchal cord. Rituximab was administered at a median of 10 days after delivery (range: 10- 33) and was continued afterwards according to the schedule. The patient previously treated with 100 mg rituximab in the 17^th^ week of gestation in pregnancy breastfed for eight weeks and did not receive treatment during lactation.

In this report, the pregnancy outcome of eight pregnancies following rituximab treatment was assessed, which led to 50% live births. No spontaneous abortions were encountered. Two patients received rituximab during pregnancy, which resulted in a healthy baby and an IUFD.

## Discussion

Disease-modifying treatment of NMOSD during pregnancy remains a controversial yet imperative decision. B cell depletion therapies have been reported in malignancies and autoimmune disorders (8, 9). There are limited studies on rituximab administration during pregnancy. In a recent case report, a new-onset NMOSD patient was treated with a total of 2g rituximab, two weeks apart, during the second trimester with a favorable outcome for the mother and the neonate (10). The baby had a normal B cell count at one month without any developmental or infectious concerns. The patient did not experience a postpartum relapse or any rituximab-related adverse events. In another study, a patient with NMOSD was treated with 1g rituximab twice, two weeks apart who had conceived three months after the last dose (11). She also received 1g rituximab at 24 weeks of pregnancy without any complications for the mother and the baby. CD19^+^B cell count was 1% at birth, which soon increased to 23% at two months. In the present study, two patients received rituximab during pregnancy (one in the 17^th^ and one in the 30^th^ week of gestation) of which the patient who received 500 mg of rituximab in the third trimester experienced IUFD due to the nuchal cord. However, this may not be attributed to rituximab administration; the general prevalence of nuchal cord ranges from 20-32% (12-14), and IUFD is reported in 0.4-5% and 0.5% of pregnancies with and without nuchal cord, respectively (12, 15). However, NMOSD may lead to pregnancy complications, especially in patients with active disease, and a recent review study has reported two stillbirths in NMOSD patients (16). 

Pregnancy has a protective effect on the disease course in multiple sclerosis patients due to hormonal changes (17); However, the same is not pertinent to NMOSD, and pregnancy does not reduce disease activity and relapse (18). Furthermore, an increase in postpartum relapses is to be expected in NMOSD patients three months after delivery; a recent study has been performed by Juto et al. also shows that there is a low rebound risk after rituximab cessation (19). Furthermore, new evidence proposes that there is a minimal secretion of rituximab in breast milk with an acceptable relative infant dose (20). A combination of the evidence suggests rituximab could be a choice in patients with NMOSD who want to be pregnant. However, it should be administered with caution and the probable adverse effects of the drug should be discussed with patients. In addition, administration of rituximab according to CD19 B cell count in pregnant patients with NMOSD may increase the efficacy and safety of this drug during the pregnancy period.

In conclusion there are limited studies evaluating the effect of rituximab in patients with NMOSD who want to be pregnant. Further studies should be done to investigate different aspects of this drug.

## Conflict of Interest:

Abdorreza Naser Moghadasi and Mohammad Ali Sahraian have received speaker’s honoraria from AryoGen Pharmed. Nassim Anjidani is the head of the medical department of Orchid Pharmed Company which is in collaboration with AryoGen Pharmed Company with respect to conducting clinical trials. The other authors have not any conflict of interest.
